# Protection against Mycobacterium ulcerans Lesion Development by Exposure to Aquatic Insect Saliva

**DOI:** 10.1371/journal.pmed.0040064

**Published:** 2007-02-27

**Authors:** Laurent Marsollier, Estelle Deniaux, Priscille Brodin, Agnès Marot, Christelle Mbondji Wondje, Jean-Paul Saint-André, Annick Chauty, Christian Johnson, Fredj Tekaia, Edouard Yeramian, Pierre Legras, Bernard Carbonnelle, Gilles Reysset, Sara Eyangoh, Geneviève Milon, Stewart T Cole, Jacques Aubry

**Affiliations:** 1 Unité de Génétique Moléculaire Bactérienne, Institut Pasteur, Paris, France; 2 Groupe d'Etude des Interactions Hôtes Pathogènes, Centre Hospitalier Universitaire et Faculté de Pharmacie d'Angers, Angers, France; 3 INSERM, Equipe Avenir, Institut Pasteur Korea, Seongbuk-gu, Seoul, Korea; 4 Laboratoire des Mycobactéries, Centre Pasteur du Cameroun, Yaoundé, Cameroun; 5 Centre de Diagnostic et de Traitement de l'Ulcère de Buruli, Pobè, Bénin; 6 Programme National de Lutte contre l'Ulcère de Buruli, Ministère de la Santé Publique, Cotonou, Bénin; 7 Unité de Génétique moléculaire des levures, Institut Pasteur, Paris, France; 8 Unité de Bio-Informatique Structurale, Institut Pasteur, Paris, France; 9 Animalerie Hospitalo-Universitaire, Angers, France; 10 Unité d'Immunophysiologie et Parasitisme Intracellulaire, Institut Pasteur, Paris, France; 11 INSERM U601, Université de Nantes, Faculté de Pharmacie, Nantes, France; Institute of Tropical Medicine, Belgium

## Abstract

**Background:**

Buruli ulcer is a severe human skin disease caused by Mycobacterium ulcerans. This disease is primarily diagnosed in West Africa with increasing incidence. Antimycobacterial drug therapy is relatively effective during the preulcerative stage of the disease, but surgical excision of lesions with skin grafting is often the ultimate treatment. The mode of transmission of this *Mycobacterium* species remains a matter of debate, and relevant interventions to prevent this disease lack (i) the proper understanding of the M. ulcerans life history traits in its natural aquatic ecosystem and (ii) immune signatures that could be correlates of protection. We previously set up a laboratory ecosystem with predatory aquatic insects of the family Naucoridae and laboratory mice and showed that (i) M. ulcerans-carrying aquatic insects can transmit the mycobacterium through bites and (ii) that their salivary glands are the only tissues hosting replicative *M. ulcerans.* Further investigation in natural settings revealed that 5%–10% of these aquatic insects captured in endemic areas have M. ulcerans–loaded salivary glands. In search of novel epidemiological features we noticed that individuals working close to aquatic environments inhabited by insect predators were less prone to developing Buruli ulcers than their relatives. Thus we set out to investigate whether those individuals might display any immune signatures of exposure to M. ulcerans-free insect predator bites, and whether those could correlate with protection.

**Methods and Findings:**

We took a two-pronged approach in this study, first investigating whether the insect bites are protective in a mouse model, and subsequently looking for possibly protective immune signatures in humans. We found that, in contrast to control BALB/c mice, BALB/c mice exposed to *Naucoris* aquatic insect bites or sensitized to *Naucoris* salivary gland homogenates (SGHs) displayed no lesion at the site of inoculation of M. ulcerans coated with *Naucoris* SGH components. Then using human serum samples collected in a Buruli ulcer–endemic area (in the Republic of Benin, West Africa), we assayed sera collected from either ulcer-free individuals or patients with Buruli ulcers for the titre of IgGs that bind to insect predator SGH, focusing on those molecules otherwise shown to be retained by M. ulcerans colonies. IgG titres were lower in the Buruli ulcer patient group than in the ulcer-free group.

**Conclusions:**

These data will help structure future investigations in Buruli ulcer–endemic areas, providing a rationale for research into human immune signatures of exposure to predatory aquatic insects, with special attention to those insect saliva molecules that bind to M. ulcerans.

## Abstract 2

Saliva from aquatic insects in areas where Buruli ulcer is endemic can protect mice against the disease's characteristic skin lesion and might play a role in natural immunity in humans.

## Editors' Summary

**Background.:**

Buruli ulcer disease is a severe skin infection caused by Mycobacterium ulcerans, a bacterium related to those that cause tuberculosis and leprosy. This poorly understood disease affects people living near slow-flowing or standing water in poor rural communities in tropical and subtropical countries. How people become infected with M. ulcerans is unclear but one possibility is that infected aquatic insects transmit it through their bites. The first sign of infection is usually a small painless swelling in the skin. Bacteria inside these swellings produce a toxin that damages nearby soft tissues until eventually the skin sloughs off to leave a large open sore. This usually heals but the resultant scar can limit limb movement. Consequently, 25% of people affected by Buruli ulcers—most of whom are children—are permanently disabled. If the disease is caught early, powerful antibiotics can prevent ulcer formation. But most patients do not seek help until the later stages when the only treatment is to cut out the infection and do a skin graft, a costly and lengthy treatment.

**Why Was This Study Done?:**

There is currently no effective way to prevent Buruli ulcers. To develop an effective preventative strategy, researchers need to determine exactly how the infection is transmitted to people and what makes some individuals resistant to infection. Previous studies have indicated that 5%–10% of some aquatic insect predators that live in areas where Buruli ulcers occur have M. ulcerans in their salivary glands and that aquatic insects carrying M. ulcerans can transmit it to mice through bites. Furthermore, people working close to water inhabited by insect predators are less likely to develop Buruli ulcers than their relatives who do not work near water. In this study, therefore, the researchers investigated whether exposure to noninfected insect saliva provides some protection against M. ulcerans infection.

**What Did the Researchers Do and Find?:**

The researchers let uninfected aquatic insects bite ten mice several times before exposing these mice and ten unbitten mice to M. ulcerans-infected water bugs. Only one pre-bitten mouse developed an M. ulcerans-containing lesion compared with eight control mice. Next, the researchers injected mice with insect salivary gland extracts before challenging them with “naked” M. ulcerans or bacteria coated with salivary gland extract. Most uninjected mice developed lesions when challenged with coated or naked M. ulcerans, as did experimental mice challenged with naked *M. ulcerans.* However, most experimental mice challenged with coated M. ulcerans remained lesion-free. In both experiments, the blood of the pre-bitten and extract-treated mice (but not the control mice) contained antibodies (immune system proteins that provide protection against infections and foreign proteins) to proteins in insect salivary gland extracts that stick to M. ulcerans. Finally, the researchers measured the blood concentration (the titer) of antibodies that bind insect salivary gland proteins in patients with Buruli ulcer and in healthy people living in the same area. People with high titers of these antibodies, they report, were less likely to have Buruli ulcers than those with low titers.

**What Do These Findings Mean?:**

These findings suggest that exposure to aquatic insect saliva may provide some protection against M. ulcerans lesion development. However, the current results have several limitations. In particular they will only be relevant to human disease if M. ulcerans is normally transmitted by insect bites, and this has not been proven yet. Also, because the human study did not measure the overall immune status of the study participants, the people with Buruli ulcers may have had a general immune deficit rather than simply lacking antibodies against insect salivary gland proteins. However, if the human findings can be repeated and expanded, they suggest that low antibody titers to salivary gland proteins might identify those people who are most susceptible to *M ulcerans* infections and who would thus benefit most from regular tests for early signs of the disease. Finally, further work on the immune mechanism by which exposure to insect salivary gland proteins protects against M. ulcerans infections may help in the development of vaccines against Buruli ulcer disease.

**Additional Information.:**

Please access these Web sites via the online version of this summary at http://dx.doi.org/10.1371/journal.pmed.0040064.

- A related *PLoS Medicine* Perspective article by Manuel T. Silva and others discusses this study and others on insect-borne parasitic diseases
- World Health Organization has information on Buruli ulcer disease
- US Centers for Disease Control and Prevention has information on Buruli ulcer
- The US Armed Forces Institute of Pathology Web site contains pages on Buruli ulcer
- Leprosy Relief Emmaus Switzerland offers information on Buruli ulcer
- Wikipedia contains pages on Buruli ulcer (note: Wikipedia is an online encyclopedia that anyone can edit)
- *PLoS Medicine* has a detailed review article on Buruli ulcer by Paul D. R. Johnson and colleagues

## Introduction

Buruli ulcer disease is now the third most common mycobacterial disease in the world, behind tuberculosis and leprosy, and its incidence in Western African countries is among the highest in the world [1,2]. This debilitating and progressive disease, characterized by skin lesions and sometimes localization in limb bones, is caused by *Mycobacterium ulcerans,* which produces a dermonecrotic toxin, a polyketide-derived macrolide called mycolactone [3,4]. Understanding the life history traits of M. ulcerans within its natural aquatic ecosystems, and the preventive and therapeutic tools for reducing the incidence of the disease are still very limited [5–7]. Currently, there is no vaccine available against Buruli ulcer. Bacillus Calmette-Guérin (BCG) vaccination provides some protection against the most severe forms of Buruli ulcer, such as osteomyelitis [8], but does not prevent most of the skin-ulcerative disease cases [9]. Initial, limited clinical evidence suggests that a rifampicin-streptomycin combination may have some beneficial effects on preulcerative lesions [10]. However, late diagnosis often results in the need for surgery [6].

The mode of M. ulcerans transmission is still unclear, with various mechanisms being suggested during the last few decades. Current evidence seems clear, however, on several points. First, with the exception of a few reports [11,12], there is no evidence of human-to-human transmission [1,2]. No disease has been observed either among health care workers in close contact with Buruli ulcer patients or among children breast-fed by mothers with Buruli ulcer lesions. Second, Buruli ulcers were shown to be significantly more prevalent in families using water from rivers than in families with access to clean water (53% and 6% respectively) or among individuals exposed to stagnant waters near the edge of hydrotelluric environments such as that along the Nile [13,14]. Third, during the late 1990s, the possibility of detecting unique genomic *IS2404* insertion sequences in M. ulcerans [15,16] allowed the extent of M. ulcerans niches in aquatic environments to be much better understood and led to a reappraisal of the most frequent and plausible mode of delivery of M. ulcerans to humans. The possible role of plants, such as the sharp-edged Echinocloa pyramidalis aquatic plant, was thus considered in Uganda [17]. In Australia, the PCR-based detection of M. ulcerans sequences showed M. ulcerans in the irrigation system of a golf course as the environmental origin of Buruli ulcer in several players or individuals living near the golf course. However, the hypothesis that M. ulcerans-loaded aerosols may have entered through barrier-disrupted skin [18] was gradually discarded.

The putative role of predatory aquatic insects as M. ulcerans vectors for humans was initially considered in 1999 [19,20] and so far, aquatic insects from the Naucoridae and Belostomatidae families have been reported as being able to be colonized by M. ulcerans and to transmit it to laboratory mice in experimental settings [21–24]. In areas endemic for Buruli ulcers, insectivorous fish were found to display M. ulcerans DNA by PCR [25]. We do not exclude other modes of transmission of M. ulcerans, but are unaware of any others that have been formally demonstrated, at least experimentally.

Field observations have shown that humans with regular activities near rivers inhabited by such aquatic insects are less prone to Buruli ulcers than are their relatives who have less exposure to this environment. Buruli ulcer epidemics in 1970 were primarily affecting displaced Rwandan refugees, with very few cases being reported in the autochthonous populations living in these environments [17]. More recently, in similar, swamp-rich environments, two of us (SE and AC) extended these observations—we found many individuals who remained Buruli ulcer–free while being regularly bitten by insects belonging to the genera *Naucoris* and *Belostoma*.

Many blood-feeding arthropods are hosts and vectors of bona fide parasitic microorganisms, including viruses, and data are accumulating that show how host and parasite interact in stable ecosystems in which the balanced fitness of each organism is reached. Ribeiro and colleagues investigated the slow- and fast-feeding behaviour of blood-feeding arthropods and compared the sialomes of the arthropods for both parasitism and adaptation to blood-feeding behaviours [26]. Much data from experimental and epidemiological studies focused on sand flies (which transmit the organisms, Leishmania spp., that cause different forms of leishmaniasis) [27,28], shows that the time taken by Phlebotomus papatasi to obtain their blood from mice is faster if the mice have been sensitized to P. papatasi saliva [29]. In addition, in mice that displayed delayed type hypersensitivity to P. papatasi saliva, no lesion developed at the site of coinoculation of Leishmania major and P. papatasi saliva [30]*.* The data generated within the context of these models prompted us to address the following questions: (i) In mice, could prior exposure to repeated bites from *M. ulcerans-*free aquatic insect predators confer some protection against *M. ulcerans-*driven pathogenic processes ? (ii) In endemic area of Buruli ulcers are any immune signatures of exposure to aquatic insect bites detectable that could distinguish Buruli ulcer-free individuals from those displaying these skin lesions?

## Methods

### Aquatic Insect Predators and Salivary Gland Homogenate

Naucoris cimicoides were collected from swamps in Western France, and Belostoma cordofana and *N. flavicollis (Macrocoris)* were from the Ouémé river in the Republic of Benin, West Africa. Insects were supplied with prey loaded with M. ulcerans (strain 01G897), as previously described [22]. For all experiments, this stably virulent strain was grown in 7H9 broth medium (Difco) supplemented with 10% Middlebrook OADC (oleic acid, dextrose, catalase; Becton-Dickinson, http://www.bd.com) and 0.05% Tween 80 (Sigma, http://www.sigmaaldrich.com) at 30 °C to the midexponential phase. The inoculum was prepared under the same conditions as previously described [21].

Accessory salivary glands were extracted from the insects with 20 mM Tris-HCl (pH 7.5) containing protease inhibitors (Complete, EDTA free; Roche Diagnostics, http://www.roche-diagnostics.com) as previously described [21]. Tissues were then homogenized by shaking with 106 μm of acid-washed glass beads (Sigma) for 5 min in a TissueLyser (Retsch, http://www.retsch.com) at 4 °C at a maximum speed. The salivary gland homogenate (SGH) consisted of the supernatant fraction recovered after debris were removed by centrifugation at 8,000 *g* for 10 min. Protein concentration of SGH was adjusted to 7 mg/ml using a method based on the Bradford dye-binding procedure (Bio-Rad, http://www.bio-rad.com) before being stored at −80 °C, or in 100% ammonium sulphate for the samples collected in Benin*.*

### Experimental Model 1: Prior Exposure of Mice to M. ulcerans-Free N. cimicoides Bites Followed by Challenge with Bites from *M. ulcerans-*Harbouring N. cimicoides

**Figure 1:**
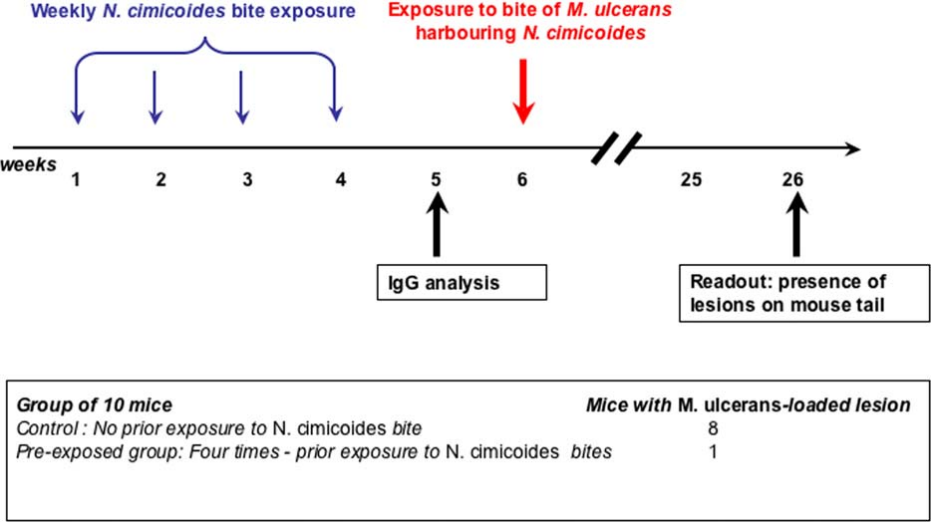
Experimental Model 1 Effect of prior exposure or not to M. ulcerans-free N. cimicoides on the development of M. ulcerans lesions after delivery of M. ulcerans from M. ulcerans-carrying *N. cimicoides.*

Six-week-old female BALB/c mice (Charles River France, http://www.criver.com/ico) were maintained under standard conditions during the experiment. The mice were first anaesthetized by intramuscular injection of ketamine (88 mg/kg) before their tails were immersed, once a week, in a *Naucoris*–containing aquarium four times for 10 s each time, to be bitten. Sera from these mice were collected at week 5 and the level of serum IgGs were monitored for binding to N. cimicoides SGH using Western blotting. Two weeks after the final exposure, mice were bitten by *M. ulcerans*–harbouring N. cimicoides and the presence of M. ulcerans in the salivary glands of insects was determined histologically as reported previously [22]. Fourteen weeks after this challenge, once inflammatory signs were observed, the mice were killed and lesion-positive tails as well as lesion-free tails were further processed to detect *M. ulcerans.* A schematic outline of the experimental setup is given in Figure 1.

### Experimental Model 2: Mouse Sensitisation with N. cimicoides SGH Followed by Subcutaneous Challenge of SGH-Treated M. ulcerans

Six-week-old female BALB/c mice were inoculated subcutaneously with 50 μg of SGH from N. cimicoides in 50 μl of PBS with fine G-26 needles once a week for four weeks. Two weeks after the last injection, the serum of each mouse was collected and the presence of N. cimicoides SGH-reactive IgGs was determined by Western blotting. At this time point, the mice were also inoculated with either M. ulcerans alone or M. ulcerans incubated with SGH. Briefly, M. ulcerans suspensions (10^6^ bacteria in 1 ml of PBS) were incubated with 1 mg of SGH, or with PBS alone as control, for 2 h at 28 °C. The bacteria were then washed three times in PBS with 0.05% Tween 80. The bacteria were then diluted in PBS to final concentrations of 10^2^ and 10^3^ bacteria/ml. One of these M. ulcerans suspensions (100 μl) was inoculated intradermally in the tail of a mouse. Three months later, all mice were killed and the number of colony-forming units (CFU) present in their tails was determined per gram of tissue, as previously described [22,31].

### Western Blot Analysis

A sample of aquatic insect SGH (60 μg) was run in an SDS-polyacrylamide gel (4%–12%) (Bio-Rad), and the separated bands were transferred onto a 0.45 μm nitrocellulose membrane (Amersham, http://www5.amershambiosciences.com). After blocking with 5% skimmed milk in PBS, the membrane was incubated with serum from mice or humans diluted 1:100 in PBS containing 0.05% Tween 80 for 1 h 30 min at 37 °C. After two washes with PBS, sheep anti-mouse IgG (heavy and light chains) peroxidase-conjugated antibodies (Interchim, http://www.interchim.com) at 0.5 μg/ml or anti-human IgG (γ chain) peroxidase-conjugated antibodies (Sigma) at 1:2,000, and 0.5 μg/ml DAB (Interchim) was used, respectively, for detecting the mouse or human IgGs bound to the different bands.

### Recovery of SGH Molecules Bound to M. ulcerans

M. ulcerans (10^7^ bacteria) were incubated with 1 mg of accessory salivary gland extract for 2 h at 28 °C with gentle agitation. The bacteria were then extensively washed and centrifuged at 5,000 *g* in PBS with 0.05% Tween 80. The resulting pellet was resuspended in 200 ml of 20 mM Tris-HCl (pH 7.5) containing protease inhibitors (Complete, EDTA-free, Roche Diagnostics). After heating at 80 °C for 1 min, the bacteria were centrifuged at 17,000 *g* for 15 min at 4 °C. The supernatant was loaded on Bis-Tris polyacrylamide gels (4%–12%) (Bio-Rad) and transferred to nitrocellulose membranes. The blots were washed and incubated with 5% skimmed milk in PBS at room temperature for 1 h and then incubated with human serum samples diluted at 1:100 in PBS containing 0.05% Tween 80. Human IgGs were detected with anti-human IgG (γ chain) peroxidase-conjugated antibodies (Sigma) at 1:2,000, and 0.5 μg/ ml DAB (Interchim).

### Detection of Aquatic Insect Saliva-Binding Immunoglobulins in Human Sera

The participants in two cohorts (“exposed” and “patients,” described below) who had given their written consent were enrolled as volunteers in this study, the protocols of which were approved by the Ministry of Health in Benin. All volunteers lived in villages near the Ouémé river, where Buruli ulcer disease is highly prevalent. Serum was prepared from 8 ml of blood from each participant and tested for potential HCV and HIV exposure using Access HIV-1/2 automated immunoassay (MDA/98/58) and Sanofi Diagnostics Pasteur Access anti-HCV automated immunoassay (plus update on five other anti-HCV assays—MDA/96/26).

The “exposed” group consisted of 55 participants (21 women and 34 men, aged 5–72 years). Typically, their professional activities were fishing and farming, and river water was used for domestic purposes. We assumed that this group had an ongoing exposure to predatory aquatic insects known to inhabit this swampy area. The “patients” group consisted of 30 Buruli ulcer patients recruited from the Centre de Diagnostic et de Traitement de l'Ulcère de Buruli in Pobè, Benin (11 women and 19 men, aged 3–74 years). Diagnoses of Buruli ulcer disease were made by Ziehl-Neelsen staining of material taken from swabs of the lesions or directly from the lesions and confirmed by PCR for M. ulcerans-specific *IS2404* DNA [16]. BCG vaccination coverage was 60% (33 of 55) for the exposed group and 63.3% (19 of 30) for the patient group.

### ELISA

Proteins (50 μg) from the insects SGH, diluted in 100 μl of PBS containing 0.05% Tween 80, were coated onto 96-well Nunc Maxisorb plates by incubation overnight at 4 °C. The coated plates were then incubated with PBS containing 5% skimmed milk at room temperature for 2 h. After three washes in PBS containing 0.05% Tween 80, the samples were incubated for 1 h at 37 °C with human serum diluted to 1:100 in PBS with 0.05% Tween 80. After three further washes, plate-bound human immunoglobulins were detected using peroxidase-conjugated goat anti-human IgG (γ chain) antibodies (Sigma) or with rat anti-human IgM (μ chain) antibodies (Interchim), and OPD (Dako, http://www.dako.com). The diluted sera were tested in triplicate and the average absorbance measured at 650 nm was expressed in optical density units.

### Statistical Analyses

The χ^2^ test was used to compare sample size distributions (proportions). One-way analysis of variance was used to compare mean values between groups followed by Newman-Keuls multiple comparison test to detect significant mean differences between pairs of groups. A *p*-value below 0.05 was considered statistically significant.

## Results

### M. ulcerans–Free N. cimicoides Bites Prevent the Development of Lesions Caused by M. ulcerans

We previously established a mouse model in which we showed that M. ulcerans-carrying aquatic bugs can transmit a few bacilli into the tails of mice exposed to the bugs for ten seconds [22]; two and four months after being bitten by M. ulcerans-carrying *N. cimicoides,* more than 80% of the mice had typical ulcerative lesions on their tails that contained M. ulcerans bacilli. In the current study, using the same mouse model we investigated the effect of prior exposure by first exposing the tails of mice to bites of M. ulcerans-free N. cimicoides and later exposing them to bites from M. ulcerans-carrying insects (Figure 1). Of the BALB/c mice previously bitten by M. ulcerans-free *N. cimicoides,* after being exposed to the mycobacterium nine out of ten did not display any lesions up to six months later when the experiment was ended. By contrast, eight out of ten of the control BALB/c mice bitten only by the M. ulcerans-carrying N. cimicoides developed typical lesions on their tails. This result suggests that prior exposure to insect bites prevented the mice from developing ulcerative lesions at sites of M. ulcerans-carrying insect bites.

**Figure 2:**
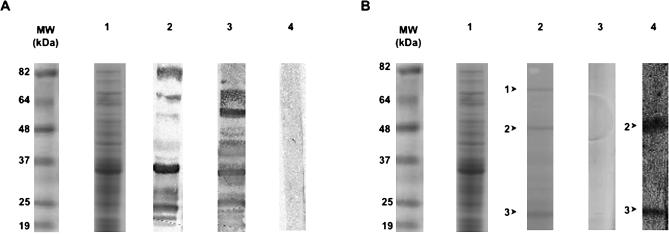
Western Blotting to Detect the Presence in Mouse Sera of IgGs Binding to N. cimicoides SGH (A) Lane 1: Coomassie staining of N. cimicoides SGH. Lane 2: Blotting of SGH with serum from mice bitten by N. cimicoides. Lane 3: Blotting of SGH with serum from mice immunized with SGH. Lane 4: Blotting of SGH with preimmune mouse serum. (B) Lane 1: Coomassie staining of N. cimicoides SGH. Lane 2: Coomassie staining of SGH-derived molecules bound to M. ulcerans cluster: arrows 1 to 3 correspond to 72, 48, and 22 kDa molecules, respectively. Lane 3: Coomassie staining of whole M. ulcerans bacteria never exposed to SGH. Lane 4: Blotting of SGH-derived molecules bound to M. ulcerans with serum of mice bitten by M. ulcerans-free N. cimicoides. MW, molecular weight.

The sera of mice exposed or not to M. ulcerans-free insect bites were screened for the presence of IgGs that bind proteins present in the aquatic insect SGH. Many proteins from the SGH bound IgGs present in the sera of mice exposed to insect bites, whereas no binding was observed with sera from mice not exposed to insect bites (Figure 2A, lane 2 versus lane 4). Three SGH proteins (22, 48, and 72 kDa) were reproducibly recovered from M. ulcerans once the latter were coincubated with SGH (Figure 2B, lane 2). Among these, two proteins (22 and 48 kDa) did bind IgGs present in the serum of mice exposed to insect bites, indicating that these insect molecules were delivered to mice during the usual biting process (Figure 2B, lane 4). Altogether, these results show that the sera of mice bitten by N. cimicoides contain IgGs that bind SGH-derived molecules, two of which are retained by M. ulcerans clusters.

We next investigated whether (i) BALB/c mice immunized with N. cimicoides SGH would not display any lesion at the site of inoculation of M. ulcerans preincubated with SGH, and (ii) any immune signature such as antibodies against the SGH molecules otherwise known to bind to M. ulcerans could be easily detected in people at risk.

### N. cimicoides SGH Prevents Skin Lesions and Results in a Lower Bacterial Load upon Infection with M. ulcerans Preincubated with SGH

Mice were first immunised with crude N. cimicoides SGH once a week for four weeks and then their tails were inoculated with M. ulcerans. As M. ulcerans multiply in the salivary glands of N. cimicoides [22], we prepared two different inocula: M. ulcerans either incubated or not with N. cimicoides SGH and then carefully washed. Twenty weeks after inoculation, control BALB/c mice (i.e., those originally given PBS instead of SGH) displayed lesions at the inoculation site. The development of lesions correlated with the number of M. ulcerans CFU present in the challenge inoculum but were independent of whether the M. ulcerans challenge inoculum had been previously incubated with SGH or not (Table 1, groups A and B). This clearly showed that the SGH proteins retained by M. ulcerans did not affect the course of the infectious and pathogenic processes in mice that had never been exposed to SGH. It further suggested that the mycobacteria delivered by M. ulcerans-carrying insects are coated with proteins from the salivary glands, which is where these bacteria multiply.

**Table 1:**
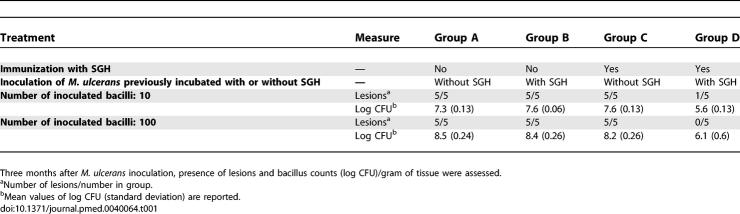
Experimental Model 2: Effect of Prior Immunization with *N. cimicoïdes* SGH on the Development of Lesions in the Tails of BALB/c Mice and the M. ulcerans Load after Subcutaneous Inoculation of M. ulcerans

Unlike the immediate transient inflammatory sign displayed by all mice upon being bitten by the insects, we saw no signs of immediate or delayed inflammation after mice were sensitized with SGH (e.g., itch, swelling, flare, or wheal). Nevertheless, serum collected from immunized mice after the last immunization (Figure 2A, lane 3)—that is, when the mice were inoculated with 10 or 100 CFU of *M. ulcerans—*contained IgGs that bound SGH-derived proteins. In contrast to the control mice, nine out of the ten mice immunized with SGH and then inoculated with M. ulcerans preincubated with SGH (Table 1, group D) did not develop lesions up to three months later. Also, in this group, the number of M. ulcerans CFU was about two orders of magnitude lower than in the three other groups (Table 1, group D versus groups A, B, and C). By contrast, mice that were immunized with SGH and then inoculated with M. ulcerans previously incubated in PBS (Table 1, group C) displayed tail lesions, suggesting that the SGH components retained by M. ulcerans do not share any common epitopes with M. ulcerans molecules. Indeed, no mouse immunoglobulin bound to any component of the insect SGH-free M. ulcerans extracts size-fractionated under the same conditions as the SGH, irrespective of the mouse serum samples tested, ruling out possible cross-reactivity with mycobacterial components (unpublished data). Altogether, these results suggest that the presence in mouse sera of IgGs that bind aquatic insect SGH-derived molecules otherwise retained by M. ulcerans clusters might be a relevant immune biomarker of their protective status. We thus investigated the potential relevance of this immune signature as a biomarker of the protective status in humans by comparing the SGH-reactive antibody profile in the sera of individuals with and without Buruli ulcers living in areas endemic for Buruli ulcers. Particular attention was paid to the SGH-derived proteins known to be retained by M. ulcerans clusters.

### In Areas Endemic for Buruli Ulcers, the Sera of Healthy Individuals Contain Higher Titres of IgGs that Bind SGH Proteins than Do the Sera of Patients with Buruli Ulcers

**Figure 3:**
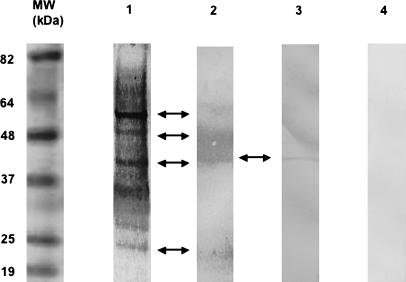
Western Blotting with Human Serum Samples Grouped According to Four Distinct N. flavicollis SGH Reactive-Antibody Profiles SGH was size-fractionated and probed with human sera as follows. Lane 1: Typical profile with serum of 49/55 (89%) exposed group participants only. Lane 2: Typical profile with serum obtained for two other members of the exposed group and one patient. Arrows correspond to protein bands at 22, 40, 48, and 54 kDa. Lane 3: Typical profile with sera obtained from two participants in each group. The arrow indicates a protein band at 40 kDa. Lane 4: No immune reactivity to SGH with serum from two members of the exposed group and from 27/30 (90%) of the patient group. MW, molecular weight.

**Figure 4:**
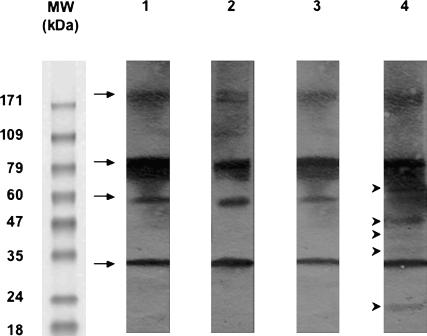
Western Blotting with Human Serum Samples as Probes for SGH Molecules that Bound to M. ulcerans N. flavicollis SGH molecules bound to *M. ulcerans* were size-fractionated and probed with human serum samples. Lane 1: M. ulcerans not preincubated with N. flavicollis SGH probed with serum from the patient group. Lane 2: M. ulcerans not preincubated with N. flavicollis SGH probed with serum from the exposed group. Lane 3: N. flavicollis SGH molecules that bind to M. ulcerans probed with serum from the patient group. Lane 4: N. flavicollis SGH molecules that bind to M. ulcerans probed with serum from the exposed group. Arrows indicate four M. ulcerans antigens. Arrow heads indicate five *M. ulcerans-*binding insect protein bands at 22, 35, 40, 48, and 66 kDa. MW, molecular weight.

All sera from individuals living in areas of Benin endemic for Buruli ulcers were first screened by Western blotting for reactive antibodies against SGH prepared from the aquatic insects present in the endemic areas, namely N. flavicollis (Figure 3). Many of the SGH constituents, such as 22, 40, 48, and 54 kDa proteins, bound IgGs present in the sera of exposed group members. Surprisingly, none of the SGH proteins bound IgGs present in 27 of the 30 sera collected from the patient group. The IgGs in the sera from the exposed group bound five of the SGH-derived molecular species (22, 35, 40, 48, and 66 kDa) shown to coat M. ulcerans (Figure 4). IgGs from the sera of participants from both groups recognised four bands (32, 56, 85, and 172 kDa) from heat-killed M. ulcerans bacteria, which are likely due to common epitopes shared with other environmental mycobacteria (Figure 4). Altogether, this qualitative analysis showed the presence of SGH-binding IgGs primarily in the sera of exposed group members.

**Figure 5:**
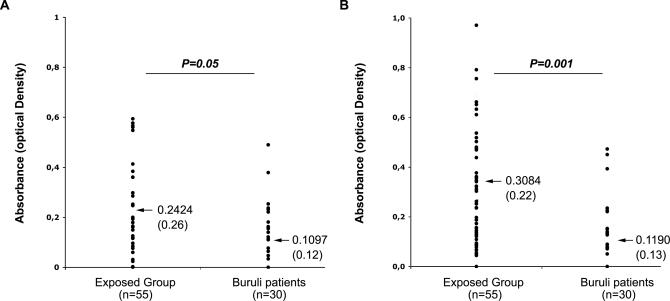
Human IgG Binding to Resident Aquatic Insect SGH by ELISA Assay Mean values are indicated by arrows and are accompanied by corresponding standard deviations (in parentheses). Results from ELISAs are shown for SGH from N. flavicollis (A) and from B. cordofana (B). Comparisons by one-way analysis of variance followed by the Newman-Keuls multiple comparison test show that the relative mean titre of specific IgG in exposed individuals is significantly higher than in patients.

We then carried out a quantitative analysis using ELISA (Figure 5). We detected SGH-reactive IgG antibodies in 87.2% of the exposed group (48/55) and 60% of the patient group (19/30). This difference was statistically significant (χ^2^ = 6.7, df = 1; *p* = 0.001). The relative mean titre of the SGH-binding IgG antibodies was significantly higher in the exposed group than in the patient group (*p* = 0.05 for N. flavicollis and *p* = 0.001 for B. cordofana [Newman-Keuls multiple comparison test]), suggesting a correlation between relative mean IgG antibody titre and the absence or presence of Buruli ulcers. Thus, in the sera of humans living in areas endemic for Buruli ulcers, the presence and titre value of antibodies that bind molecules derived from aquatic insect salivary glands may be potentially relevant immune biomarkers of a protective status in the absence of any preulcerative or ulcerative lesions containing *M. ulcerans.*

## Discussion

Using a previously developed mouse model [21,22], in this study we demonstrated (i) that preulcerative and ulcerative stages of Buruli ulcer at sites of inoculation of M. ulcerans are prevented if the mice have been bitten by M. ulcerans-free insects before being exposed to insects harbouring M. ulcerans, and (ii) that the presence of insect saliva–reactive antibodies in the mouse serum is an immune signature that correlates with this protective status. However, this protective status does not prevent M. ulcerans from establishing themselves and expanding, although the size of the bacterial population is one to two orders of magnitude lower than in control mice not previously exposed to insect bites and displaying a lesion at the site of inoculation.

In addition, we wished to determine whether insect saliva-reactive IgGs could be used as an immune signature that correlates with protection from disease. Two cohorts of human study participants in the Buruli ulcer–endemic area of Benin—those without ulcers (but assumed exposed to insect bites) and those with ulcers (actual Buruli ulcer patients)—were screened for the titre of IgGs that bind to insect predator SGH, focusing on those molecules shown to be retained by M. ulcerans clusters. In this investigation we established that, when present, IgG titres were lower in the Buruli ulcer patient group than in the Buruli ulcer-free individual group.

Buruli ulcer is the most common mycobacterial disease in immunocompetent humans after tuberculosis and leprosy. Although BCG can prevent M. ulcerans–driven lesion development in a mouse model [32], no evidence of a protective effect of routine BCG vaccination against Buruli ulcer was detected in a case-control study in Benin [9]. Prior sensitization to environmental *Mycobacterium* species may partly explain the failure of BCG in African countries, as observed for tuberculosis [33]. A classical *Mycobacterium*-based vaccine is thus unlikely to induce the proper immune effectors/regulators that would prevent bacterial colonisation and the skin-damaging processes in the M. ulcerans-containing site. This problem led us to investigate vaccine strategies that have already proved promising against microorganisms transmitted by arthropod hosts and vectors [34–36]. Furthermore, it has been shown that some molecules present in the saliva of the blood-feeding arthropods that are hosts and vectors of certain parasites are immunogenic and may help prevent infestation by the parasites causing the pathogenesis. For example, a protective effect was reported in mice bitten by L. major-free blood-feeding P. papatasi sand flies [37] or in mice immunized with P. papatasi saliva molecules as either SGH or saliva-encoding DNA sequences [28,30].

In our mouse model [22] we established that nine out of ten BALB/c mice exposed to M. ulcerans-free N. cimicoides bites or immunised with N. cimicoides SGH did not develop tail lesions at the sites of M. ulcerans delivery either by the bite of M. ulcerans-loaded N. cimicoides or by a syringe containing in vitro–grown M. ulcerans colonies that had been incubated with SGH. Unlike in the *Leishmania* model, in which the delivery of parasites by L. major-carrying sand flies to naive mice considerably enhanced the severity of the lesion [37,38], we did not observe any marked difference between mice that received either M. ulcerans with SGH by syringe or bites of insects whose salivary glands contained M. ulcerans. Our data show that in naive mice, both inoculation methods initiate similar pathogenic processes to those seen for SGH-treated or saliva-free M. ulcerans. Similar protection was obtained whether a low- or a medium-dose inoculum was given. However, despite the absence of any lesion, a high mycobacterial load was recovered from mouse tails previously exposed to SGH. This suggests that when M. ulcerans is coated with SGH or delivered from the *Naucoris* salivary gland, mice that have an immune response against SGH or the saliva molecules covering the bacteria present a skin microenvironment that may delay the production of tissue-damaging mycolactone toxin or other mycobacterial molecules. This would allow the tissue-protective immune effectors to be properly balanced with bacteria-targeted immune effectors.

In *M. ulcerans–*positive but lesion-free mice given M. ulcerans treated with SGH-derived molecules, the presence of IgGs that bind insect molecules—and more specifically the SGH molecules coating *M. ulcerans*—may be an interesting biomarker to focus on when investigating the presence of protective effectors/regulators that remodel the early niche of *M. ulcerans.* It is indeed reasonable to investigate whether both type 1 T and regulatory T lymphocytes might be activated by saliva-derived peptides loading the major histocompatibility complex molecules displayed by dendritic leucocytes. Indeed, in the experimental L. major/mouse model, the protection conferred by SGH is coupled to activation of saliva-reactive CD4^+^ cells, a positive skin delayed-type hypersensitivity to SGH, and CD4-dependent interferon-γ production [37]. Protection studies using the purified protein antigenic molecules, and adoptive transfer experiments of saliva-reactive antibodies and/or saliva-reactive CD4^+^ T lymphocytes will determine whether this is the case in our experimental system.

Although the seroepidemiological data collected from humans living in Benin in an area endemic for Buruli ulcers are only preliminary, they require some further comments. We detected a higher relative mean antibody titre to SGH from local aquatic insects in the sera from Buruli ulcer-free healthy participants (“exposed” group) than in the sera of Buruli ulcer patients (“patients” group) from the same endemic areas. The healthy individuals regularly work and live around the aquatic areas and probably are regularly exposed to aquatic insect bites. This situation is similar to that described for many residents living in regions endemic for Lyme disease. Those experiencing a persistent tick-associated itch were less likely to develop Lyme disease than those who did not experience this reaction [35]. Many different immune signatures to tick components, including salivary gland antigenic molecules, may prevent *Borrelia* disease or other diseases driven by parasitic microorganisms transmitted by ticks [36]. A similar situation has been extensively documented in an area endemic for leishmaniasis, in which the occurrence of *Leishmania-*caused lesions decreased with age and level of antibodies against salivary gland molecules in the absence of any parasite-targeted serological signature [39].

Our results suggest potentially important immune markers for epidemiological studies in regions in which Buruli ulcer is showing features of an emerging disease. Comprehensive studies that integrate the monitoring of aquatic insect saliva–reactive antibodies and T lymphocytes would allow patients who do not have high titres of antibodies to SGH to benefit from regular tests for early signs of the disease that can be cured with appropriate antibiotics. Such studies have been recently set up in other endemic areas or in emerging areas such as Cameroon.

As well as being the first report, to our knowledge, of a robust, experimental mouse model that closely mimics the processes driven by M. ulcerans in humans in endemic areas, our study also shows, we believe for the first time, that effective protection against M. ulcerans-caused pathogenic processes is possible but that this will require immunogens derived from insect host saliva rather than from pathogenic microorganisms. However, investigations into the mechanisms that prevent Buruli ulcer formation are still needed. These studies should address whether the aquatic insect SGH–reactive antibodies play a role in protection or whether they are only relevant biomarkers of M. ulcerans delivery by one of their hosts and vectors. Our results are the first step to determine the immune mechanisms that prevent M. ulcerans development into the nodular and ulcerative stages—the Buruli ulcers—in humans. They will help structure future investigations in Buruli ulcer endemic area, providing a rationale for considering at least two novel parameters: (i) the presence of immune signatures of exposure to insect predators, and (ii) the insect saliva molecules that bind to *M. ulcerans.*

## Supporting Information

Alternative Language Abstract S1 Translation of the Abstract into French by Laurent Marsollier and Colleagues (21 KB DOC)

## Abbreviations

BCG: bacillus Calmette-Guérin
CFU: colony-forming unit(s)
SGH: salivary gland homogenate
