# Supplementary material for: Protection against Mycobacterium ulcerans Lesion Development by Exposure to Aquatic Insect Saliva
Source: PLoS Med. 2007 Feb 27;4(2):e64. doi: 10.1371/journal.pmed.0040064 (PMC1808094; doi:10.1371/journal.pmed.0040064)
Supplement: Alternative Language Abstract S1 — (21 KB DOC) [file pmed.0040064.sd001.doc]

L’ulcère de Buruli, maladie essentiellement cutanée causée par *Mycobacterium ulcerans,* est devenue la troisième mycobactériose chez le sujet immunocompétent. L’antibiothérapie qui est efficace sur les formes précoces doit être associée le plus souvent à la chirurgie. A l’heure actuelle il n’existe pas de vaccin spécifique efficace contre l’ulcère de Buruli. Pendant de nombreuses années, il a été suggéré que le BCG pouvait avoir une efficacité, mais des études récentes viennent de prouver l’inefficacité du BCG. Le développement de nouvelles stratégies vaccinales est donc indispensable afin de lutter contre l’ulcère de Buruli.

Concernant le mode de Transmission de *M. ulcerans* on connaît peu de chose. Le seul vecteur identifié à ce jour est la punaise aquatique. Lors d’études en zones endémiques, nous avons noté que les pêcheurs ou les personnes ayant un contact important avec les zones aquatiques où prolifèrent les punaises ne présentent pas de lésion à *M. ulcerans*. Nous avons donc émis l’hypothèse suivante : Les personnes se faisant régulièrement piquer par des punaises saines sont protégées contre l’établissement de lésions à *M. ulcerans*.

Utilisant notre modèle expérimental, nous avons montré que des souris sensibilisées à la piqûre d’insectes sains étaient protégées contre le développement de lésions. De plus, les animaux sensibilisés développent une immunité humorale dirigée contre des constituants salivaires ayant la propriété de se fixer au bacille.

La seconde partie de notre travail a consisté à réaliser une étude sérologique au Bénin. Il est apparu que les sujets présentant des lésions à *M. ulcerans* ont un taux relatif IgG dirigés contre les constituants du suc salivaire bien inférieur à celui des personnes vivant dans les mêmes zones mais qui sont régulièrement exposées aux piqûres des punaises.

L’identification des molécules responsables de cet effet protecteur est nécessaire afin d’envisager de nouvelles stratégies protectrices en zones endémiques.
